# PL-VIO: Tightly-Coupled Monocular Visual–Inertial Odometry Using Point and Line Features

**DOI:** 10.3390/s18041159

**Published:** 2018-04-10

**Authors:** Yijia He, Ji Zhao, Yue Guo, Wenhao He, Kui Yuan

**Affiliations:** 1 Institute of Automation, Chinese Academy of Sciences, Beijing 100190, China; guoyue2013@ia.ac.cn (Y.G.); wenhao.he@ia.ac.cn (W.H.); kui.yuan@ia.ac.cn (K.Y.); 2 University of Chinese Academy of Sciences, Beijing 100049, China; 3 The ReadSense Ltd., Shanghai 200040, China; zhaoji84@gmail.com

**Keywords:** sensor fusion, visual–inertial odometry, tightly-coupled, point and line features

## Abstract

To address the problem of estimating camera trajectory and to build a structural three-dimensional (3D) map based on inertial measurements and visual observations, this paper proposes point–line visual–inertial odometry (PL-VIO), a tightly-coupled monocular visual–inertial odometry system exploiting both point and line features. Compared with point features, lines provide significantly more geometrical structure information on the environment. To obtain both computation simplicity and representational compactness of a 3D spatial line, Plücker coordinates and orthonormal representation for the line are employed. To tightly and efficiently fuse the information from inertial measurement units (IMUs) and visual sensors, we optimize the states by minimizing a cost function which combines the pre-integrated IMU error term together with the point and line re-projection error terms in a sliding window optimization framework. The experiments evaluated on public datasets demonstrate that the PL-VIO method that combines point and line features outperforms several state-of-the-art VIO systems which use point features only.

## 1. Introduction

Localization and navigation have attracted much attention in recent years with respect to a wide range of applications, particularly for self-driving cars, service robots, and unmanned aerial vehicles, etc. Several types of sensors are utilized for localization and navigation, such as global navigation satellite systems (GNSSs) [1], laser lidar [2,3], inertial measurement units (IMUs), and cameras [4,5]. However, they have obvious respective drawbacks: GNSSs only provide reliable localization information if there is a clear sky view [6]; laser lidar suffers from a reflection problem for objects with glass surfaces [7]; measurements from civilian IMUs are noisy, such that inertial navigation systems may drift quickly due to error accumulation [8]; and monocular simultaneous localization and mapping (SLAM) can only recover the motion trajectory up to a certain scale and it tends to be lost when the camera moves fast or illumination changes dramatically [9,10,11]. As a result, sensor fusion methods, especially for visual–inertial navigation systems, have drawn widespread attention [12]. The acceleration and angular velocity information from an IMU can significantly improve the monocular SLAM system [13,14]. Furthermore, both IMUs and cameras are light-weight and low-cost, and as such they are widely used in civilian applications.

According to directly or indirectly fused measurements from sensors, visual–inertial odometry (VIO) systems can be divided into two main streams: loosely-coupled and tightly-coupled approaches. Loosely-coupled approaches [15,16] process images and IMU measurements by two estimators that estimate relative motion separately and fuse the estimates from two estimators to obtain the final result. Tightly-coupled approaches [17,18] use one estimator to find optimal estimates by fusing raw measurements from the camera and IMU directly. Compared to loosely-coupled approaches, tightly-coupled approaches are generally more accurate and robust. In this paper, the proposed PL-VIO method is a tightly-coupled VIO system. Related works on tightly-coupled VIO approaches can be categorized by the number of linearizations in the measurement model [14]. These approaches based on the extended Kalman filter (EKF) process a measurement only once in the updating step, while batch nonlinear optimization approaches linearize multiple times during the optimization step. Filtering-based approaches [19,20] integrate IMU measurements to propagate/predict the state, and then update/correct the latest state with visual measurements. Since the coordinates of three-dimensional (3D) landmarks are included in the state vector, the computational complexity of the EKF increases quadratically with the number of landmarks. To address this problem, Mourikis and Roumeliotis [21] proposed the multi-state constraint Kalman filter (MSCKF) which marginalizes out the landmark coordinates from the state vector. A drawback of this method is that the landmark measurements used to update the state need to be moved out of view of the camera, which means that not all the current visual measurements are used in the filter. Furthermore, the linearization errors make the filter inconsistent [14].

Optimization-based approaches obtain the optimal estimate by minimizing a joint nonlinear cost function with IMU measurement residuals and visual re-projection residuals. Thus, optimization-based approaches can repeat the linearization of a state vector at different points to achieve higher accuracy than filtering-based methods [14]. The IMU measurement constraints are computed by integrating IMU measurements between frames. However, the standard IMU integration method is closely connected with the initial IMU body state at the first frame. When the estimated state changes, all integrated IMU measurements need to be re-calculated. Lupton and Sukkarieh [22] proposed an IMU pre-integration technology which avoids such repeated integrations. IMU pre-integration has been widely used in optimization-based VIO [18,23,24]. Forster et al. [14] reformulated the IMU pre-integration by treating the rotation group on a manifold instead of using Euler angles. Liu et al. [13] proposed the continuous pre-integration method. Although optimization-based approaches have achieved high accuracy, computation becomes expensive with more and more landmarks being added into the optimization. OKVIS [18] used a first-in-last-out sliding window method for bound computation by marginalizing the measurements from the oldest state. Shen et al. [23] proposed a two-way marginalization to selectively marginalize the body state and landmarks.

Although significant achievements have been made in the VIO area, most VIO systems only use the point features as the visual information. However, point detection in textureless environments and point tracking in illumination-changing scenes are challenging [25,26]. In contrast, line segments are a proper alternative solution in these scenes. Additionally, line segments provide more structural information on the environment than points [27]. For visual-only SLAM, there are several works combining point and line features to estimate camera motion [28,29]. The simplest way to integrate line features in a SLAM system is to use two endpoints to represent the line. Matching the endpoints of a line from different views is difficult. Furthermore, the 3D spatial line only has four degrees-of-freedom (DoFs), while two 3D endpoints introduce six parameters, which results in over-parameterization. Bartoli and Sturm [30] proposed the orthonormal representation, which uses a three-DoF rotation matrix and a one-DoF rotation matrix to update the line parameters during optimization. The orthonormal representation has been used in some stereo visual SLAM systems [27,31]. For VIO approaches, Kottas and Roumeliotis [26] investigated the observability of the VIO using line features only. Kong et al. [25] built a stereo VIO system combining point and line features by utilizing trifocal geometry. However, these works involve filtering-based VIO. In our proposed PL-VIO method, we integrate line features into the optimization framework in order achieve higher accuracy than filtering-based methods.

To build a structural 3D map and obtain the camera’s motion, we propose the PL-VIO system, which optimizes the system states by jointly minimizing the IMU pre-integration constraints together with the point and line re-projection errors in sliding windows. Compared to the traditional methods which only use point features, our method utilizes the additional line feature, aiming to increase the robustness and accuracy in an illumination-changing environment. Our main contributions are as follows:

- To the best of our knowledge, the proposed PL-VIO is the first optimization-based monocular VIO system using both points and lines as landmarks.
- To tightly and efficiently fuse the information from visual and inertial sensors, we introduce a sliding window model with IMU pre-integration constraints and point/line features. To represent a 3D spatial line compactly in optimization, the orthonormal representation for a line is employed. All the Jacobian matrices of error terms with respect to IMU body states are derived for solving the sliding window optimization efficiently.
- We compare the performances of the proposed PL-VIO with three state-of-the-art monocular VIO methods including ROVIO [17], OKVIS [18], and VINS-Mono [32] on both the EuRoc dataset and the PennCOSYVIO dataset, for which detailed evaluation results are reported.

The remainder of this paper is organized as follows. First, we describe the mathematical preliminaries in Section 2, and then formulate the sliding window-based visual–inertial fusion method in Section 3. Next, we describe our PL-VIO system and implementation details in Section 4. Section 5 shows the experimental results. Finally, conclusions and potential future works are given in Section 6.

## 2. Mathematical Formulation

### 2.1. Notations

**Figure 1:**
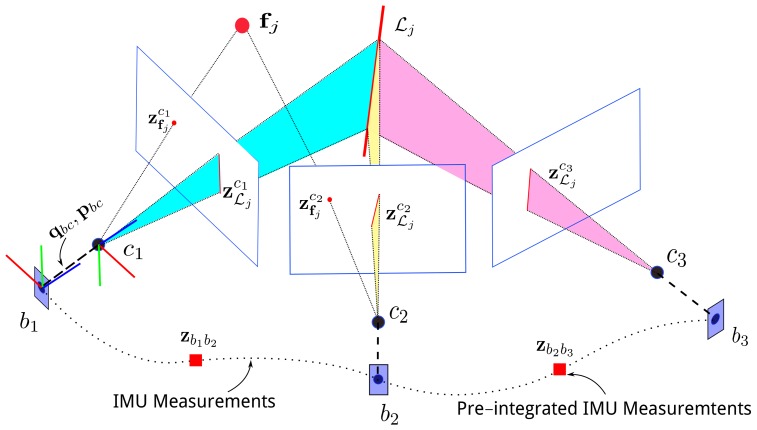
An illustration of visual–inertial sensors, point observations, and line observations. IMU: inertial measurement unit.

Figure 1 illustrates the visual–inertial sensors, and the visual observations for point and line features. We denote $c_{i}$ as the camera frame at time t=i and $b_{i}$ as the IMU body frame at the same time. *w* is the Earth’s inertial frame. ${\left(\cdot \right)}^{c}$ means the vector (·) is expressed in frame *c*. Quaternion $q_{xy}$ is used to rotate a vector from frame *y* to frame *x*, and the corresponding matrix form is $R_{xy}$. We use $p_{xy}$ to translate a vector from frame *y* to frame *x*. Quaternion $q_{bc}$ and vector $p_{bc}$ are the extrinsic parameters between the camera frame and the body frame, and these extrinsic parameters are known in the provided datasets or calibrated with the Kalibr calibration toolbox [33]. $f_{j}$ and $L_{j}$ are the *j*th point landmark and the line landmark, respectively, in the map. z represents a measurement; specifically $z_{f_{j}}^{c_{i}}$ is the *j*th point feature observed by *i*th camera frame, and $z_{b_{i}b_{j}}$ represents a pre-integrated IMU measurement between two keyframes.

### 2.2. IMU Pre-Integration

A 6-axis IMU, including a 3-axis accelerometer and a 3-axis gyroscope, can measure the acceleration a and the angular velocity ω of the body frame with respect to the inertial frame [14]. The raw measurements $\hat{\omega }$ and $\hat{a}$ are affected by bias and white noise:

(1) $$\begin{array}{cc} {\hat{\omega }}^{b} & =\omega^{b}+b_{g}^{b}+n_{g}^{b} \end{array}$$

(2) $$\begin{array}{cc} {\hat{a}}^{b} & =R_{bw}\left(a^{w}+g^{w}\right)+b_{a}^{b}+n_{a}^{b} \end{array}$$

where $b_{g}^{b},b_{a}^{b}$ and $n_{g}^{b},n_{a}^{b}$ are the biases and white noises from gyroscope and accelerometer, respectively. $g^{w}={\left[0,0,g\right]}^{⊤}$ is the gravity vector in frame *w*. We use the following kinematics for IMU-driven system [34]:

(3) $${\dot{p}}_{wb_{t}}=v_{t}^{w},\;{\dot{v}}_{t}^{w}=a_{t}^{w},\;{\dot{q}}_{wb_{t}}=q_{wb_{t}}⊗\left[\begin{array}{c} 0\\ \frac{1}{2}\omega^{b_{t}} \end{array}\right]$$

where ⊗ denotes the quaternion multiplication operation.

Given the IMU body state at time t=i, namely $p_{wb_{i}},v_{i}^{w},q_{wb_{j}}$, and series values of ω and a during the duration t∈[i,j], we can obtain the body state at time t=j by integrating Equation (3):

(4) $$\begin{array}{cc} p_{wb_{j}} & =p_{wb_{i}}+v_{i}^{w}\Delta t+\int \int_{t\in [i,j]}\left(R_{wb_{t}}a^{b_{t}}-g^{w}\right)\delta t^{2}\\ v_{j}^{w} & =v_{i}^{w}+\int_{t\in [i,j]}\left(R_{wb_{t}}a^{b_{t}}-g^{w}\right)\delta t\\ q_{wb_{j}} & =\int_{t\in [i,j]}q_{wb_{t}}⊗\left[\begin{array}{c} 0\\ \frac{1}{2}\omega^{b_{t}} \end{array}\right]\delta t \end{array}$$

where Δt is the time difference between *i* and *j*. In Equation (4), the body state propagation starts from the *i*th frame $b_{i}$. When the state of $b_{i}$ is changed, we need to re-propagate all the measurements. Since body states are adjusted at each iteration during the optimization, Equation (4) is time-consuming. By decomposing $q_{wb_{j}}$ to $q_{wb_{i}}⊗q_{b_{i}b_{t}}$, Equation (4) can be written as:

(5) $$\begin{array}{cc} p_{wb_{j}} & =p_{wb_{i}}+v_{i}^{w}\Delta t-\frac{1}{2}g^{w}\Delta t^{2}+R_{wb_{i}}\alpha_{b_{i}b_{j}}\\ v_{j}^{w} & =v_{i}^{w}-g^{w}\Delta t+R_{wb_{i}}\beta_{b_{i}b_{j}}\\ q_{wb_{j}} & =q_{wb_{i}}⊗q_{b_{i}b_{j}} \end{array}$$

where

(6) $$\begin{array}{cc} \alpha_{b_{i}b_{j}} & =\int \int_{t\in [i,j]}\left(R_{b_{i}b_{t}}a^{b_{t}}\right)\delta t^{2}\\ \beta_{b_{i}b_{j}} & =\int_{t\in [i,j]}\left(R_{b_{i}b_{t}}a^{b_{t}}\right)\delta t\\ q_{b_{i}b_{j}} & =\int_{t\in [i,j]}q_{b_{i}b_{t}}⊗\left[\begin{array}{c} 0\\ \frac{1}{2}\omega^{b_{t}} \end{array}\right]\delta t \end{array}$$

$z_{b_{i}b_{j}}={\left[\alpha_{b_{i}b_{j}},\beta_{b_{i}b_{j}},q_{b_{i}b_{j}}\right]}^{⊤}$ are called pre-integration measurements [22] and can be calculated directly without knowing the body states of $b_{i}$, which means when body state is changed the re-propagation is not necessary. We treat the pre-integrated measurements as constraint factors between successive keyframes.

The pre-integration model (Equation (6)) is derived from continuous time and neglects the biases and noises. In practice, IMU measurements are collected from discrete times, and noise should be considered. In this work, we use mid-point integration to integrate the IMU measurements. The IMU body propagation using measurements at discrete moments *k* and k+1 is calculated by:

(7) $$\begin{array}{cc} \hat{\omega } & =\frac{1}{2}\left(\left({\hat{\omega }}^{b_{k}}-b_{g}^{b_{k}}+n_{g}^{b_{k}}\right)+\left({\hat{\omega }}^{b_{k+1}}-b_{g}^{b_{k}}+n_{g}^{b_{k+1}}\right)\right)\\ {\hat{q}}_{b_{i}b_{k+1}} & ={\hat{q}}_{b_{i}b_{k}}⊗\left[\begin{array}{c} 1\\ \frac{1}{2}\hat{\omega }\delta t \end{array}\right]\\ \hat{a} & =\frac{1}{2}\left(R_{b_{i}b_{k}}\left({\hat{a}}^{b_{k}}-b_{a}^{b_{k}}+n_{a}^{b_{k}}\right)+R_{b_{i}b_{k+1}}\left({\hat{a}}^{b_{k+1}}-b_{a}^{b_{k+1}}+n_{g}^{b_{k+1}}\right)\right)\\ {\hat{\alpha }}_{b_{i}b_{k+1}} & ={\hat{\alpha }}_{b_{i}b_{k}}+{\hat{\beta }}_{b_{i}b_{k}}\delta t+\frac{1}{2}\hat{a}\delta t^{2}\\ {\hat{\beta }}_{b_{i}b_{k+1}} & ={\hat{\beta }}_{b_{i}b_{k}}+\hat{a}\delta t \end{array}$$

At the beginning, k=i, we have $q_{b_{i}b_{i}}={\left[0,0,0,1\right]}^{⊤}$, and $\alpha_{b_{i}b_{i}},\beta_{b_{i}b_{i}}$ are zero vectors. In Equation (7), in order to compute the pre-integration measurements efficiently, we assume biases are constant between two keyframes:

(8) $$b_{a}^{b_{k}}=b_{a}^{b_{k+1}},\;b_{g}^{b_{k}}=b_{g}^{b_{k+1}},\;k\in \left[i,j-1\right]$$

In practice, biases change slowly. We model biases with random walk noises:

(9) $$b_{a}^{b_{k+1}}=b_{a}^{b_{k}}+n_{b_{a}}\delta t,\;b_{g}^{b_{k+1}}=b_{g}^{b_{k}}+n_{b_{g}}\delta t$$

where the Gaussian white noises are defined as $n_{b_{a}}\in N\left(0,\sigma_{b_{a}}^{2}\right)$ and $n_{b_{g}}\in N\left(0,\sigma_{b_{g}}^{2}\right)$. When bias changes with a small increments, instead of computing pre-integrated measurements iteratively, we use a first-order approximation to update ${\hat{q}}_{b_{i}b_{j}},{\hat{\alpha }}_{b_{i}b_{j}},{\hat{\beta }}_{b_{i}b_{j}}$ [14]:

(10) $$\begin{array}{cc} {\hat{\alpha }}_{b_{i}b_{j}} & \leftarrow {\hat{\alpha }}_{b_{i}b_{j}}+J_{b_{a}^{i}}^{\alpha }\delta b_{a}^{b_{i}}+J_{b_{g}^{i}}^{\alpha }\delta b_{g}^{b_{i}}\\ {\hat{\beta }}_{b_{i}b_{j}} & \leftarrow {\hat{\beta }}_{b_{i}b_{j}}+J_{b_{a}^{i}}^{\beta }\delta b_{a}^{b_{i}}+J_{b_{g}^{i}}^{\beta }\delta b_{g}^{b_{i}}\\ {\hat{q}}_{b_{i}b_{j}} & \leftarrow {\hat{q}}_{b_{i}b_{j}}⊗\left[\begin{array}{c} 1\\ \frac{1}{2}J_{b_{g}^{i}}^{q}\delta b_{g}^{b_{i}} \end{array}\right] \end{array}$$

where $J_{b_{i}^{a}}^{\alpha }=\frac{\partial \alpha_{b_{i}b_{j}}}{\partial \delta b_{a}^{b_{i}}},\;J_{b_{g}^{i}}^{\alpha }=\frac{\partial \alpha_{b_{i}b_{j}}}{\partial \delta b_{g}^{b_{i}}},\;J_{b_{a}^{i}}^{\beta }=\frac{\partial \beta_{b_{i}b_{j}}}{\partial \delta b_{a}^{b_{i}}},\;J_{b_{g}^{i}}^{\beta }=\frac{\partial \beta_{b_{i}b_{j}}}{\partial \delta b_{g}^{b_{i}}},\;J_{b_{g}^{i}}^{q}=\frac{\partial q_{b_{i}b_{j}}}{\partial \delta b_{g}^{b_{i}}}$ are the Jacobian matrices of pre-integrated measurements with respect to bias at time *i*. They can be derived with error state transformation matrices, as shown in Appendix A. The covariance matrix of pre-integrated measurements $\Sigma_{b_{i}b_{j}}$ can be computed iteratively with IMU propagation, and more details are provided in Appendix A.

### 2.3. Geometric Representation of Line

A straight line only has four DoFs. Thus, the compact parameterization of a straight line is with four parameters. In our system, we treat a straight line in 3D space as an infinite line and adopt two parameterizations for a 3D line as in [27]. Plücker line coordinates consisting of six parameters are used for transformation and projection due to their simplicity. An orthonormal representation consisting of four parameters is used for optimization due to its compactness.

#### 2.3.1. Plücker Line Coordinates

**Figure 2:**
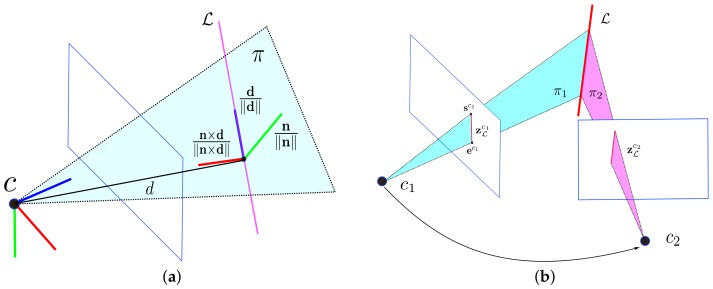
Plücker coordinates for line features. (**a**) Plücker line coordinates; (**b**) Initialization of a newly observed line.

In Figure 2a, a 3D spatial line L in Plücker coordinates is represented by $L={\left(n^{⊤},d^{⊤}\right)}^{⊤}\in R^{6}$, where $d\in R^{3}$ is the line direction vector, and $n\in R^{3}$ is the normal vector of the plane determined by the line and the coordinate origin. The Plücker coordinates are over-parameterized since there is an implicit constraint between the vector n and d, i.e., $n^{⊤}d=0$. Therefore, the Plücker coordinates can not be directly used in unconstrained optimization. However, with a 3D line represented by a normal vector and a direction vector it is simple to perform triangulation from two views geometrically, and it is also convenient to model the line geometry transformation.

For line geometry transformation, given the transformation matrix $T_{cw}=\left[\begin{array}{cc} R_{cw} & p_{cw}\\ 0 & 1 \end{array}\right]$ from the world frame *w* to the camera frame *c*, we can transform the Plücker coordinates of a line by [30]

(11) $$L^{c}=\left[\begin{array}{c} n^{c}\\ d^{c} \end{array}\right]=T_{cw}L_{w}=\left[\begin{array}{cc} R_{cw} & {\left[p_{cw}\right]}_{\times }R_{cw}\\ 0 & R_{cw} \end{array}\right]L^{w}$$

where ${\left[\cdot \right]}_{\times }$ is the skew-symmetric matrix of a three-dimensional vector, and $T_{cw}$ is the transform matrix used to transform a line from frame *w* to frame *c*.

When a new line landmark is observed in two different camera views, the Plücker coordinates are easily calculated. As shown in Figure 2b, the 3D line L is captured by cameras $c_{1}$ and $c_{2}$ as $z_{L}^{c_{1}}$ and $z_{L}^{c_{2}}$, respectively. The line segment $z_{L}^{c_{1}}$ in the normalized image plane can be represented by two endpoints, $s^{c_{1}}={\left[u_{s},v_{s},1\right]}^{⊤}$ and $e^{c_{1}}={\left[u_{e},v_{e},1\right]}^{⊤}$. Three non-collinear points, including two endpoints of a line segment and the coordinate origin $C={\left[x_{0},y_{0},z_{0}\right]}^{⊤}$, determine a plane $\pi ={\left[\pi_{x},\pi_{y},\pi_{z},\pi_{w}\right]}^{⊤}$ in 3D space:

(12) $$\pi_{x}\left(x-x_{0}\right)+\pi_{y}\left(y-y_{0}\right)+\pi_{z}\left(z-z_{0}\right)=0$$

where

(13) $$\left[\begin{array}{c} \pi_{x}\\ \pi_{y}\\ \pi_{z} \end{array}\right]={\left[s^{c_{1}}\right]}_{\times }e^{c_{1}},\;\pi_{w}=\pi_{x}x_{0}+\pi_{y}y_{0}+\pi_{z}z_{0}$$

Given the two plane $\pi_{1}$ and $\pi_{2}$ in camera frame $c_{1}$, the dual Plücker matrix $L^{*}$ can be computed by

(14) $$L^{*}=\left[\begin{array}{cc} {\left[d\right]}_{\times } & n\\ -n^{⊤} & 0 \end{array}\right]=\pi_{1}\pi_{2}^{⊤}-\pi_{2}\pi_{1}^{⊤}\in R^{4\times 4}$$

The Plücker coordinates L in camera frame $c_{1}$ are easily extracted from the dual Plücker matrix $L^{*}$. It can be seen that n and d do not need to be unit vectors.

#### 2.3.2. Orthonormal Representation

Since 3D spatial lines only have four DoFs, the orthonormal representation (U,W)∈SO(3) × SO(2) is more suitable than Plücker coordinates during optimization. Additionally, the orthonormal representation and Plücker coordinates can be converted from each other, which means we can adopt both of them in a SLAM system for different purposes. In this section, we will introduce the details of orthonormal representation. As shown in Figure 2a, a coordinate system is defined on the 3D line. The normalized normal vector and the normalized direction vector are the two axes of the coordinate system. The third axis is determined by crossing the other two axes vectors. We can define the rotation matrix between the line coordinate and the camera frame as U:

(15) $$\begin{array}{c} U=R\left(\psi \right)=\left[\begin{array}{ccc} \frac{n}{∥n∥} & \frac{d}{∥d∥} & \frac{n\times d}{∥n\times d∥} \end{array}\right] \end{array}$$

where $\psi ={\left[\psi_{1},\psi_{2},\psi_{3}\right]}^{⊤}$ consists of the rotation angles around the *x*-, *y*-, and *z*-axes of a camera frame. The relationship between the Plücker coordinates and U is:

(16) $$\left[\begin{array}{cc} n & d \end{array}\right]=\left[\begin{array}{ccc} \frac{n}{∥n∥} & \frac{d}{∥d∥} & \frac{n\times d}{∥n\times d∥} \end{array}\right]\left[\begin{array}{cc} ∥n∥ & 0\\ 0 & ∥d∥\\ 0 & 0 \end{array}\right]$$

Since the combination of $\left(∥n∥,∥d∥\right)$ in Equation (16) only has one DoF, we can use trigonometric functions to represent it:

(17) $$\begin{array}{c} W=\left[\begin{array}{cc} \cos(\phi ) & -\sin(\phi )\\ \sin(\phi ) & \cos(\phi ) \end{array}\right]=\frac{1}{\sqrt{\left({∥n∥}^{2}+{∥d∥}^{2}\right)}}\left[\begin{array}{cc} ∥n∥ & -∥d∥\\ ∥d∥ & ∥n∥ \end{array}\right] \end{array}$$

where ϕ is a rotation angle. Recall that the distance from coordinate origin to the 3D line is $d=\frac{∥n∥}{∥d∥}$, so W contains the information about the distance *d*. According to the definitions of U and W, these four DoFs include three DoFs from the rotation matrix, which transforms the line coordinate to the camera frame, and one DoF from the distance *d*. We use $O={\left[\psi ,\phi \right]}^{⊤}$ as the minimal representation of a 3D spatial line during optimization.

Once a 3D line L has been optimized with the orthonormal representation, the corresponding Plücker coordinates for the line can be computed by:

(18) $$L^{\prime }={\left[w_{1}u_{1}^{T},w_{2}u_{2}^{T}\right]}^{T}=\frac{1}{\sqrt{\left({∥n∥}^{2}+{∥d∥}^{2}\right)}}L$$

where $u_{i}$ is the *i*th column of matrix U, $w_{1}=\cos\left(\phi \right)$, and $w_{2}=\sin\left(\phi \right)$. There is a scale factor between $L^{\prime }$ and L, but they represent the same 3D spatial line.

## 3. Tightly-Coupled Visual–Inertial Fusion

**Figure 3:**
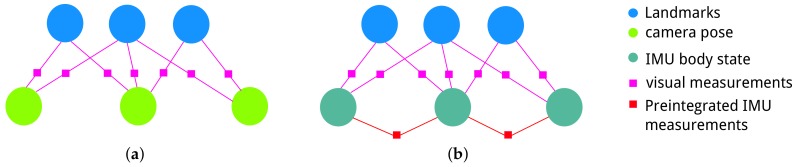
Factor graphs of (**a**) the visual simultaneous localization and mapping (SLAM) problem versus (**b**) visual–inertial SLAM.

In visual SLAM, bundle adjustment is used to optimize the camera poses and 3D map points by minimizing the re-projection error in image planes. Bundle adjustment by nonlinear optimization can be treated as a factor graph [35] as shown in Figure 3a: round nodes are the camera poses or 3D landmarks needed to be optimized; edges with square boxes represent the visual measurements as constraints between nodes. For visual–inertial fusion, we can also use the tightly-coupled graph-based framework to optimize all the state variables of the visual–inertial system [23]. As shown in Figure 3b, the factor graph not only contains the visual measurements, but also takes the pre-integrated IMU measurements as edges to constrain the successive IMU body states.

### 3.1. Sliding Window Formulation

In order to achieve both computation efficiency and high accuracy, we use the sliding window formulation for factor graph optimization. The full state variables in a sliding window at time *i* are defined as:

(19) $$\begin{array}{cc} X & ={\left[x_{n},x_{n+1},\ldots ,x_{n+N},\lambda_{m},\lambda_{m+1},\ldots ,\lambda_{m+M},O_{o},O_{o+1},\ldots ,O_{o+O}\right]}^{⊤}\\ x_{i} & ={\left[p_{wb_{i}},q_{wb_{i}},v_{i}^{w},b_{a}^{b_{i}},b_{g}^{b_{i}}\right]}^{⊤},i\in \left[n,n+N\right] \end{array}$$

where $x_{i}$ is described by the *i*th IMU body position, velocity, and orientation in the world frame, and biases in the IMU body frame. Subscripts n,m, and *o* are the start indexes of body states, point landmarks, and line landmarks, respectively. *N* is the number of keyframes in the sliding window. *M* and *O* are the numbers of point landmarks and line landmarks observed by all keyframes in the sliding window, respectively. We only use one variable, the inverse depth $\lambda_{k}$, to parameterize the *k*th point landmark from its first observed keyframe. $O_{l}$ is the orthonormal representation of the *l*th line feature in the world frame *w*.

We optimize all the state variables in the sliding window by minimizing the sum of cost terms from all the measurement residuals:

(20) $$\begin{array}{cc} \min_{X}\rho \left({∥r_{p}-J_{p}X∥}_{\Sigma_{p}}^{2}\right) & +\sum_{i\in B}\rho \left({∥r_{b}\left(z_{b_{i}b_{i+1}},X\right)∥}_{\Sigma_{b_{i}b_{i+1}}}^{2}\right)\\ & +\sum_{(i,j)\in F}\rho \left({∥r_{f}\left(z_{f_{j}}^{c_{i}},X\right)∥}_{\Sigma_{f_{j}}^{c_{i}}}^{2}\right)+\sum_{(i,l)\in L}\rho \left({∥r_{l}\left(z_{L_{i}}^{c_{i}},X\right)∥}_{\Sigma_{L_{i}}^{c_{i}}}^{2}\right) \end{array}$$

where $r_{b}\left(z_{b_{i}b_{i+1}},X\right)$ is an IMU measurement residual between the body state $x_{i}$ and $x_{i+1}$. *B* is the set of all pre-integrated IMU measurements in the sliding window. $r_{f}\left(z_{f_{j}}^{c_{i}},X\right)$ and $r_{l}\left(z_{L_{i}}^{c_{i}},X\right)$ are the point feature re-projection residual and the line feature re-projection residual, respectively. *F* and *L* are the sets of point features and line features observed by camera frames. $\left\{r_{p},J_{p}\right\}$ is prior information that can be computed after marginalizing out a frame in the sliding window [23], and $J_{p}$ is the prior Jacobian matrix from the resulting Hessian matrix after the previous optimization. ρ is the Cauchy robust function used to suppress outliers.

We use Levenberg–Marquard algorithm to solve the nonlinear optimization problem (20). The optimal state estimates X can be found by iteratively update from an initial guess $X_{0}$ as:

(21) $$X_{t+1}^{\prime }=X_{t}⊕\delta X$$

where ⊕ is the operator used to update parameters with increment δX. For position, velocity, bias, and inverse depth, the update operator and increments δ are easily defined:

(22) $$p^{\prime }=p+\delta p,\;v^{\prime }=v+\delta v,\;\lambda^{\prime }=\lambda +\delta \lambda ,\;b^{\prime }=b+\delta b$$

However, the update operator and increments δ for state attitude q are more complicated. Four parameters are used in quaternion to represent the three-DoF rotation, so it is over-parameterized. The increment for rotation should only be three-dimensional. Similar to [18], we use a perturbation $\delta \theta \in R^{3}$ in the tangent space as the rotation increment. Thus, rotation q can be updated by the quaternion multiplication:

(23) $$q^{\prime }=q⊗\delta q,\;\delta q\approx \left[\begin{array}{c} 1\\ \frac{1}{2}\delta \theta \end{array}\right]$$

We can also write it as a rotation matrix form:

(24) $$R^{\prime }\approx R\left(I+{\left[\delta \theta \right]}_{\times }\right)$$

where I is a 3×3 identity matrix. Similarly, we can update the orthonormal representation as:

(25) $$\begin{array}{cc} U^{\prime } & \approx U(I+{\left[\delta \psi \right]}_{\times })\\ W^{\prime } & \approx W\left(I+\left[\begin{array}{cc} 0 & -\delta \phi \\ \delta \phi & 0 \end{array}\right]\right) \end{array}$$

The increment for the orthonormal representation is $\delta O={\left[{\left[\delta \psi \right]}_{\times },\delta \phi \right]}^{⊤}$. Finally, the increment δX during the optimization can be defined as:

(26) $$\begin{array}{cc} \delta X & ={\left[\delta x_{n},\delta x_{n+1},\ldots ,\delta x_{n+N},\delta \lambda_{m},\delta \lambda_{m+1},\ldots ,\delta \lambda_{m+M},\delta O_{o},\delta O_{o+1},\ldots ,\delta O_{o+O}\right]}^{⊤}\\ \delta x_{i} & ={\left[\delta p,\delta \theta ,\delta v,\delta b_{a}^{b_{i}},\delta b_{g}^{b_{i}}\right]}^{⊤},i\in \left[n,n+N\right] \end{array}$$

At each iteration, the increment δX can be solved by Equation (20):

(27) $$\left(H_{p}+H_{b}+H_{f}+H_{l}\right)\delta X=\left(b_{p}+b_{b}+b_{f}+b_{l}\right)$$

where $H_{p},H_{b},H_{f}$, and $H_{l}$ are the Hessian matrices for prior residuals, IMU measurement residuals, and point and line re-projection residuals, respectively. For residual $r_{\left(\cdot \right)}$, we have $H_{\left(\cdot \right)}=J_{\left(\cdot \right)}^{⊤}\Sigma_{\left(\cdot \right)}^{-1}J_{\left(\cdot \right)}$ and $b_{\left(\cdot \right)}=-J_{\left(\cdot \right)}^{⊤}\Sigma_{\left(\cdot \right)}^{-1}r_{\left(\cdot \right)}$, where $J_{\left(\cdot \right)}$ is the Jacobian matrix of residuals vector $r_{\left(\cdot \right)}$ with respect to δX, and $\Sigma_{\left(\cdot \right)}$ is the covariance matrix of measurements. Formulations of residuals and Jacobian matrices will be defined in the following subsections.

### 3.2. IMU Measurement Model

Since the pre-integrated IMU measurement computed by Equation (10) is a constraint factor between two successive keyframes $b_{i}$ and $b_{j}$, the IMU measurement residual can be defined as:

(28) $$r_{b}\left(z_{b_{i}b_{j}},X\right)=\left[\begin{array}{c} r_{p}\\ r_{\theta }\\ r_{v}\\ r_{ba}\\ r_{bg} \end{array}\right]={\left[\begin{array}{c} R_{b_{i}w}\left(p_{wb_{j}}-p_{wb_{i}}-v_{i}^{w}\Delta t+\frac{1}{2}g^{w}\Delta t^{2}\right)-{\hat{\alpha }}_{b_{i}b_{j}}\\ 2{\left[{\hat{q}}_{b_{j}b_{i}}⊗\left(q_{b_{i}w}⊗q_{wb_{j}}\right)\right]}_{xyz}\\ R_{b_{i}w}\left(v_{j}^{w}-v_{i}^{w}+g^{w}\Delta t\right)-{\hat{\beta }}_{b_{i}b_{j}}\\ b_{a}^{b_{j}}-b_{a}^{b_{i}}\\ b_{g}^{b_{j}}-b_{g}^{b_{i}} \end{array}\right]}_{15\times 1}$$

where ${\left[\cdot \right]}_{xyz}$ extracts the real part of a quaternion which is used to approximate the three-dimensional rotation error [18].

During the nonlinear optimization, the Jacobian matrix of the IMU measurement residual with respect to the body state $x_{i}$ and $x_{j}$ is computed by:

(29) $$\begin{array}{cc} J_{b} & =\left[\begin{array}{cc} \frac{\partial r_{b}}{\partial \delta x_{i}} & \frac{\partial r_{b}}{\partial \delta x_{j}} \end{array}\right]\\ \frac{\partial r_{b}}{\partial \delta x_{i}} & ={\left[\begin{array}{ccccc} -R_{b_{i}w} & {\left[R_{b_{i}w}\left(p_{wb_{j}}-p_{wb_{i}}-v_{i}^{w}\Delta t+\frac{1}{2}g^{w}\Delta t^{2}\right)\right]}_{\times } & -R_{b_{i}w}\Delta t & -J_{b_{a}^{i}}^{\alpha } & -J_{b_{g}^{i}}^{\alpha }\\ 0 & {\left\lfloor -{\left[q_{wb_{j}}^{-1}⊗q_{wb_{i}}\right]}_{L}{\left[{\hat{q}}_{b_{i}b_{j}}\right]}_{R}\right\rfloor }_{3\times 3} & 0 & 0 & J_{b_{g}^{i}}^{r_{\theta }}\\ 0 & {\left[R_{b_{i}w}\left(v_{j}^{w}-v_{i}^{w}+g^{w}\Delta t\right)\right]}_{\times } & -R_{b_{i}w} & -J_{b_{a}^{i}}^{\beta } & -J_{b_{g}^{i}}^{\beta }\\ 0 & 0 & 0 & -I & 0\\ 0 & 0 & 0 & 0 & -I \end{array}\right]}_{15\times 15}\\ \frac{\partial r_{b}}{\partial \delta x_{j}} & ={\left[\begin{array}{ccccc} -R_{b_{i}w} & 0 & 0 & 0 & 0\\ 0 & {\left\lfloor -{\left[{\hat{q}}_{b_{i}b_{j}}^{-1}⊗q_{wb_{i}}^{-1}⊗q_{wb_{j}}\right]}_{L}\right\rfloor }_{3\times 3} & 0 & 0 & 0\\ 0 & 0 & R_{b_{i}w} & 0 & 0\\ 0 & 0 & 0 & I & 0\\ 0 & 0 & 0 & 0 & I \end{array}\right]}_{15\times 15} \end{array}$$

where ${\left[q\right]}_{L}$ and ${\left[q\right]}_{R}$ are the left- and right- quaternion-product matrices, respectively [36]. The operator ${\left\lfloor \cdot \right\rfloor }_{3\times 3}$ is used to extract a 3×3 matrix from the bottom right block of (·). The Jacobian matrix is calculated by $J_{b_{g}^{i}}^{r_{\theta }}=\frac{\partial r_{\theta }}{\partial \delta b_{g}^{b_{i}}}={\left\lfloor -{\left[q_{wb_{j}}^{-1}⊗q_{wb_{i}}⊗q_{b_{i}b_{j}}\right]}_{L}\right\rfloor }_{3\times 3}J_{b_{g}^{i}}^{q}$.

### 3.3. Point Feature Measurement Model

For point features, the re-projection error of a 3D point is the image distance between the projected point and the observed point. In this work, we deal with the point and line feature measurements in the normalized image plane. Given the *k*th point feature measurement at frame $c_{j}$, $z_{f_{k}}^{c_{j}}={\left[u_{f_{k}}^{c_{j}},v_{f_{k}}^{c_{j}},1\right]}^{⊤}$, the re-projection error is defined as:

(30) $$\begin{array}{cc} r_{f}\left(z_{f_{k}}^{c_{i}},X\right) & =\left[\begin{array}{c} \frac{x^{c_{j}}}{z^{c_{j}}}-u_{f_{k}}^{c_{j}}\\ \frac{y^{c_{j}}}{z^{c_{j}}}-v_{f_{k}}^{c_{j}} \end{array}\right]\\ f_{k}^{c_{j}} & =\left[\begin{array}{c} x^{c_{j}}\\ y^{c_{j}}\\ z^{c_{j}} \end{array}\right]=R_{bc}^{⊤}\left(R_{wb_{j}}^{⊤}\left(R_{wb_{i}}\left(\left(R_{bc}\frac{1}{\lambda_{k}}\left[\begin{array}{c} u_{f_{k}}^{c_{i}}\\ v_{f_{k}}^{c_{i}}\\ 1 \end{array}\right]+p_{bc}\right)+p_{wb_{i}}\right)-p_{wb_{j}}\right)-p_{bc}\right) \end{array}$$

where $z_{f}^{c_{i}}={\left[u_{f_{k}}^{c_{i}},v_{f_{k}}^{c_{i}},1\right]}^{⊤}$ is the first observation of the feature in camera frame $c_{i}$, and the inverse depth of the feature $\lambda_{k}$ is also defined in camera frame $c_{i}$.

In order to minimize the point’s re-projection error (30), we need to optimize the rotation and the position of frame $b_{i},b_{j}$, and the feature inverse depth λ. The corresponding Jacobian matrix can be obtained by the chain rule:

(31) $$J_{f}=\frac{\partial r_{f}}{\partial f^{c_{j}}}\left[\begin{array}{ccc} \frac{\partial f^{c_{j}}}{\partial x_{i}} & \frac{\partial f^{c_{j}}}{\partial x_{j}} & \frac{\partial f^{c_{j}}}{\partial \delta \lambda } \end{array}\right]$$

With

(32) $$\begin{array}{cc} \frac{\partial r_{f}}{\partial f^{c_{j}}} & =\left[\begin{array}{ccc} \frac{1}{z^{c_{j}}} & 0 & -\frac{x^{c_{j}}}{{\left(z^{c_{j}}\right)}^{2}}\\ 0 & \frac{1}{z^{c_{j}}} & -\frac{y^{c_{j}}}{{\left(z^{c_{j}}\right)}^{2}} \end{array}\right]\\ \frac{\partial f^{c_{j}}}{\partial x_{i}} & ={\left[\begin{array}{ccccc} R_{bc}^{⊤}R_{wb_{j}}^{⊤} & -R_{bc}^{⊤}R_{wb_{j}}^{⊤}R_{wb_{i}}{\left[f^{b_{i}}\right]}_{\times } & 0 & 0 & 0 \end{array}\right]}_{3\times 15}\\ \frac{\partial f^{c_{j}}}{\partial x_{j}} & ={\left[\begin{array}{ccccc} -R_{bc}^{⊤}R_{wb_{j}}^{⊤} & R_{bc}^{T}{\left[f^{b_{j}}\right]}_{\times } & 0 & 0 & 0 \end{array}\right]}_{3\times 15}\\ \frac{\partial f^{c_{j}}}{\partial \delta \lambda } & =-\frac{1}{\lambda }R_{bc}^{⊤}R_{wb_{j}}^{⊤}R_{wb_{i}}R_{bc}f_{c_{i}} \end{array}$$

where $f^{b_{i}}$ is the 3D point vector in the *i*th IMU body frame. We define the covariance matrix of point measurement $\Sigma_{f_{k}}^{c_{i}}$ as a 2×2 diagonal matrix by assuming that the detected point features have pixel noise on both the vertical and horizontal directions in the image plane.

### 3.4. Line Feature Measurement Model

The re-projection error of a line measurement is defined as the distance from endpoints to the projected line. For a pin-hole model camera, a 3D spatial line $L={\left[n,d\right]}^{⊤}$ can be projected to the camera image plane by [27]:

(33) $$l=\left[\begin{array}{c} l_{1}\\ l_{2}\\ l_{3} \end{array}\right]=Kn^{c}=\left[\begin{array}{ccc} f_{y} & 0 & 0\\ 0 & f_{x} & 0\\ -f_{y}c_{x} & -f_{x}c_{y} & f_{x}f_{y} \end{array}\right]n^{c}$$

where K is the projection matrix for a line feature. When projecting a line to the normalized image plane, K is an identity matrix. From the projection Equation (33), the coordinates of the line segment projected by a 3D line are only related with the normal vector n.

Given a 3D line $L_{l}^{w}$ and the orthonormal presentation $O_{l}$ in a world frame, we firstly transform it to camera frame $c_{i}$ by Equation (11). Then, we project it to the image plane to get the projection line $l_{l}^{c_{i}}$. The re-projection error in camera frame *i* is defined as

(34) $$r_{l}\left(z_{L_{l}}^{c_{i}},X\right)=\left[\begin{array}{c} d(s_{l}^{c_{i}},l_{l}^{c_{i}})\\ d(e_{l}^{c_{i}},l_{l}^{c_{i}}) \end{array}\right]$$

With d(s,l) indicating the distance function from endpoint s to the projection line l:

(35) $$d\left(s,l\right)=\frac{s^{⊤}l}{\sqrt{l_{1}^{2}+l_{2}^{2}}}$$

The *i*th body state and *l*th line parameters are optimized by minimizing the line re-projection error $r_{l}\left(z_{L_{l}}^{c_{i}}\right)$. The corresponding Jacobian matrix can be obtained by the chain rule:

(36) $$J_{l}=\frac{\partial r_{l}}{\partial l^{c_{i}}}\frac{\partial l^{c_{i}}}{\partial L^{c_{i}}}\left[\begin{array}{cc} \frac{\partial L^{c_{i}}}{\partial \delta x^{i}} & \frac{\partial L^{c_{i}}}{\partial L^{w}}\frac{\partial L^{w}}{\partial \delta O} \end{array}\right]$$

With

(37) $$\begin{array}{cc} \frac{\partial r_{l}}{\partial l^{c_{i}}} & ={\left[\begin{array}{ccc} \frac{-l_{1}{\left(s_{l}^{c_{i}}\right)}^{⊤}l}{{\left(l_{1}^{2}+l_{2}^{2}\right)}^{\left(\frac{3}{2}\right)}}+\frac{u_{s}}{{\left(l_{1}^{2}+l_{2}^{2}\right)}^{\left(\frac{1}{2}\right)}} & \frac{-l_{2}{\left(s_{l}^{c_{i}}\right)}^{⊤}l}{{\left(l_{1}^{2}+l_{2}^{2}\right)}^{\left(\frac{3}{2}\right)}}+\frac{v_{s}}{{\left(l_{1}^{2}+l_{2}^{2}\right)}^{\left(\frac{1}{2}\right)}} & \frac{1}{{\left(l_{1}^{2}+l_{2}^{2}\right)}^{\left(\frac{1}{2}\right)}}\\ \frac{-l_{1}{\left(e_{l}^{c_{i}}\right)}^{⊤}l}{{\left(l_{1}^{2}+l_{2}^{2}\right)}^{\left(\frac{3}{2}\right)}}+\frac{u_{e}}{{\left(l_{1}^{2}+l_{2}^{2}\right)}^{\left(\frac{1}{2}\right)}} & \frac{-l_{2}{\left(e_{l}^{c_{i}}\right)}^{⊤}l}{{\left(l_{1}^{2}+l_{2}^{2}\right)}^{\left(\frac{3}{2}\right)}}+\frac{v_{e}}{{\left(l_{1}^{2}+l_{2}^{2}\right)}^{\left(\frac{1}{2}\right)}} & \frac{1}{{\left(l_{1}^{2}+l_{2}^{2}\right)}^{\left(\frac{1}{2}\right)}} \end{array}\right]}_{2\times 3}\\ \frac{\partial l^{c_{i}}}{\partial L^{c_{i}}} & ={\left[\begin{array}{cc} K & 0 \end{array}\right]}_{3\times 6}\\ \frac{\partial L^{c}}{\partial \delta x^{i}} & ={\left[\begin{array}{ccccc} T_{bc}^{-1}\left[\begin{array}{c} R_{wb}^{⊤}{\left[d^{w}\right]}_{\times }\\ 0_{3\times 3} \end{array}\right] & T_{bc}^{-1}\left[\begin{array}{c} {\left[R_{wb}^{⊤}\left(n^{w}+{\left[d^{w}\right]}_{\times }p_{wb}\right)\right]}_{\times }\\ R_{wb}^{⊤}d^{w}{]}_{\times } \end{array}\right] & 0 & 0 & 0 \end{array}\right]}_{6\times 15}\\ \frac{\partial L^{c_{i}}}{\partial L^{w}}\frac{\partial L^{w}}{\partial \delta O} & =T_{wc}^{-1}{\left[\begin{array}{cccc} 0 & -w_{1}u_{3} & w_{1}u_{2} & -w_{2}u_{1}\\ w_{2}u_{3} & 0 & -w_{2}u_{1} & w_{1}u_{2} \end{array}\right]}_{6\times 4} \end{array}$$

The derivation details are provided in Appendix B. Similar to the point measurement covariance matrix, the covariance matrix of line measurement $\Sigma_{L_{l}}^{c_{i}}$ is defined by assuming the endpoints of a line segment have pixel noise.

## 4. Monocular Visual Inertial Odometry with Point and Line Features

**Figure 4:**
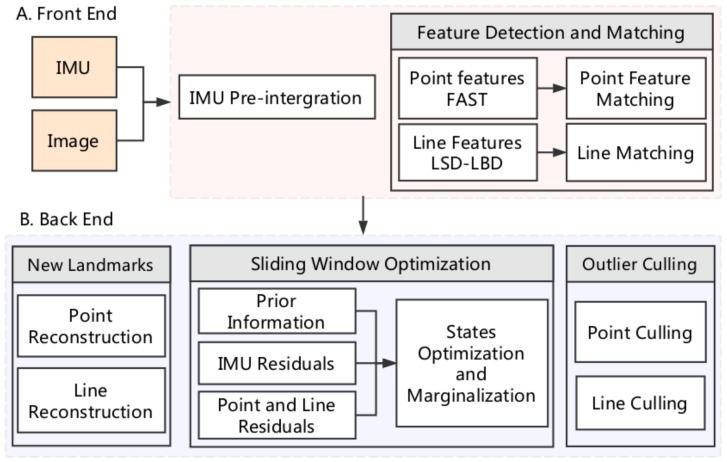
Overview of our point–line visual–inertial odometry (PL-VIO) system. A is the front end module is used to extract information from the raw measurements; B is the back end module is used to estimate and optimize the body states.

As shown in Figure 4, the proposed PL-VIO system has two main modules: the front end and the back end. The front-end module is used to pre-process the measurements from IMU and camera. The back-end module is used to estimate and optimize the body states. We will introduce the details in the following subsections.

### 4.1. Front End

The front end extracts information from the raw measurements of the camera and IMU. The body state is updated by propagation with each new IMU measurement, and the newest body state is used as the initial value in sliding window optimization. Additionally, the new IMU measurements are pre-integrated to constrain the successive IMU body states during optimization.

As for image processing, the point and line features are detected in two separate threads. When a new frame comes, the point features are tracked from the previous frame to the new frame by the KLT optical flow algorithm [37]. Then, we use the RANSAC framework with an essential matrix test to remove outliers. If the number of tracked point features is less than a threshold after outlier rejection, new corner features which are detected by the FAST detector [38] will be added. As to the line features, line segments in new frame are detected by the LSD detector [39] and matched with those in the previous frame by the appearance-based descriptor LBD [40]. We use geometric constraints to remove outliers of line matches. For example, the distance between the midpoints of two matched lines should be no more than $\delta_{dist}^{th}$ pixels, and the angle difference should be no more than $\delta_{angle}^{th}$ degrees. After the feature detection and matching, the point features and the endpoints of line segments are projected onto the normalized image plane. Additionally, a frame is selected as a new keyframe if the average parallax of the tracked point features is larger than a threshold.

### 4.2. Back End

In the back-end thread, the points and lines are firstly triangulated to build re-projection residuals. In order to get good landmark estimations, the inverse depth of a point feature is estimated with all the observations. For line triangulation, we only choose two frames with the furthest spatial distance in the sliding window to initialize the Plücker coordinates.

After we get the initial estimation of map points/lines and the IMU body state predicted from IMU measurements, the sliding window optimization described in Section 3 is used to find the optimal states. To limit the size of the state vector X, a two-way marginalization strategy is adopted to remove states from the sliding window [23]. When the second newest frame $x_{n+N-1}$ is a keyframe, we marginalize out the earliest frame $x_{n}$ with all its measurements. Otherwise, if the second newest frame is not a keyframe, we discard the visual measurements from this frame and reserve its IMU measurements in the pre-integration measurements. New prior information is gained based on the marginalized measurements, reserving the constraint information from the removed states.

Lastly, we cull the invalid map points and lines. If the inverse depth of a point is negative, we will delete this point from the map. If the re-projection residuals of a line exceed a threshold it will be removed from the map.

### 4.3. Implementation Details

To bootstrap the VIO system, we adopt the visual–inertial alignment method [41] to recover the scale, gravity vector, initial biases, and velocity of the IMU initial body state. The sliding window is with N=10 keyframes. Each frame has at most 150 point features, and 150 line segments. The thresholds used in line matching are set with $\delta_{dist}^{th}=60$ pixels and $\delta_{angle}^{th}=30$${}^{\circ }$. Since the visual–inertial system has only four unobservable DoFs (the yaw direction and the absolute position), the optimization methods for six DoFs may introduce illusory information into the roll and pitch directions by automatically taking steps along these directions to minimize the cost function. After the sliding window optimization, we reset the body state by rotating it back with the increments along the roll and pitch directions. All the numerical optimizations are solved using the Levenberg–Marquardt method in the Ceres solver library [42]. The line detection and matching codes are provided by OpenCV 3 [43].

## 5. Experimental Results

We evaluated our PL-VIO system using two public benchmark datasets: the EuRoc MAV Dataset [44] and the PennCOSYVIO Dataset [45].The accuracy of the PL-VIO method is compared with that of three state-of-the-art monocular VIO methods to validate the advantages of the PL-VIO method: ROVIO [17] and OKVIS [18] in monocular mode, and VINS-Mono [32] without loop closure. ROVIO is a tightly-coupled form of VIO based on the extended Kalman filter (EKF). It directly uses the intensity errors from images to find the optimal state during the update step. OKVIS is a sliding window optimization algorithm with point features which can work with monocular or stereo modes. VINS-Mono is a complete VIO-SLAM system employing point features to optimize IMU body states in a sliding window, and performs loop closure. All of the experiments were performed on the computer with an Intel Core i7-6700HQ CPU with 2.60 GHz, 16 GB RAM, and the ROS Kinetic [46].

### 5.1. EuRoc MAV Dataset

The EuRoc micro aerial vehicle (MAV) datasets were collected by an MAV in two indoor scenes, which contain stereo images from a global shutter camera at 20FPS and synchronized IMU measurements at 200 Hz [44]. Each dataset provides a ground truth trajectory given by the VICON motion capture system. All the extrinsic and intrinsic parameters are also provided in the datasets. In our experiments, we only used the images from the left camera.

**Figure 5:**
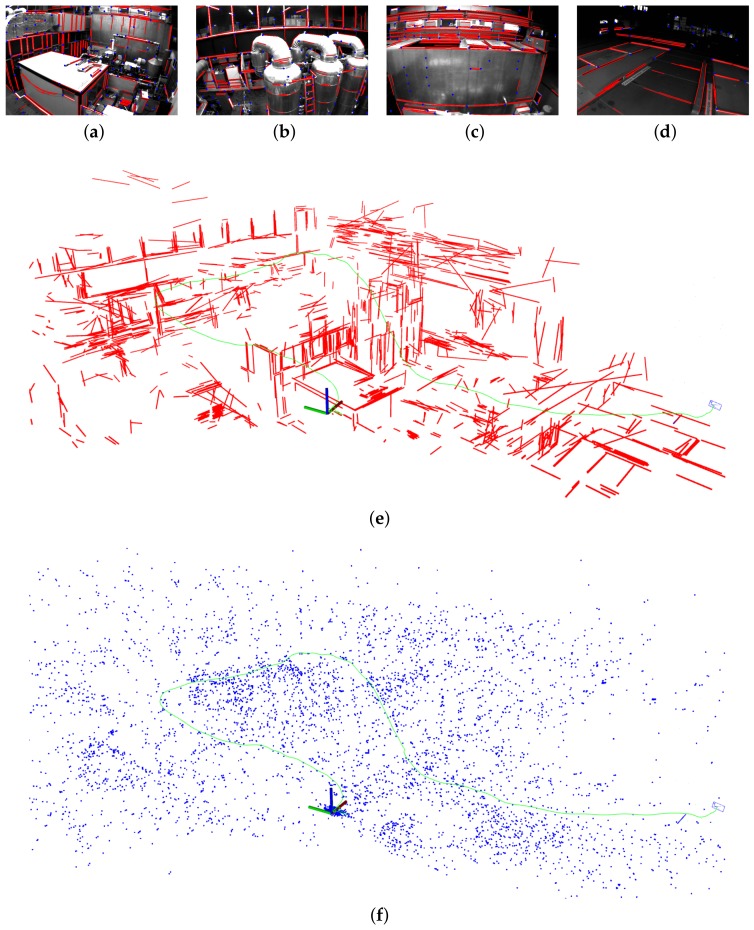
Mapping results in the MH_05_difficult sequence. (**a**–**d**) Detected point and line features for different frames. (**e**) The reconstructed line map (red) and the trajectory (green). The scenes with rich line features (such as the window, baluster, and cabinet) are clearly reconstructed from the map. (**f**) The reconstructed point map (blue). As compared to the line map, the structure is hard to identify in the point map.

The main advantage of line features is that they provide significant geometry structure information with respect to the environment. As an example, we show the reconstructed map built by PL-VIO from the MH_05_difficult sequence in Figure 5. The four images in Figure 5a–d were captured by an MAV flying in a machine hall, which showed the room’s structure. As shown in Figure 5d, the line segment detection in the weak illumination scene was more robust than point feature detection. From the reconstructed 3D map, it can be seen that the geometry of the environment is described by the line segments, and thus semantic information could be extracted from the map. This is useful for robot navigation.

**Figure 6:**
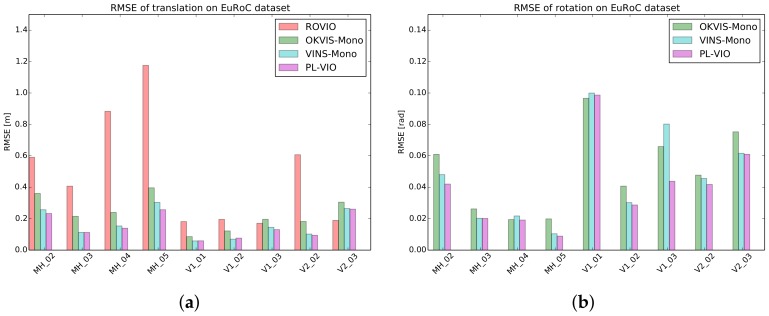
RMSEs for ROVIO, OKVIS-Mono, and Vins-Mono without loop closure, and for the proposed PL-VIO using the EuRoc dataset. (**a**) RMSEs in translation. (**b**) RMSEs in rotation. The rotation errors of ROVIO are one order of magnitude higher than those of other three methods, so its result is not reported in (**b**).

For quantitative evaluation, we compared our PL-VIO with three state-of-the-art monocular visual–inertial methods: ROVIO [17], OKVIS [18] in monocular mode, and VINS-Mono [32] without loop closure. For the fair comparison, default parameters provided by the authors of these comparison methods were used. We chose the absolute pose error (APE) as the evaluation metric, which directly compared the trajectory error between the estimate and the ground truth [47]. The open-source package evo (https://michaelgrupp.github.io/evo/) provides an easy-to-use interface to evaluate the trajectories of odometry and SLAM algorithms. We employed this tool to evaluate these methods in this section. Table 1 shows the root mean square error (RMSE) of translation and rotation along all the trajectory, and their histograms are also provided as shown in Figure 6.

**Figure 7:**
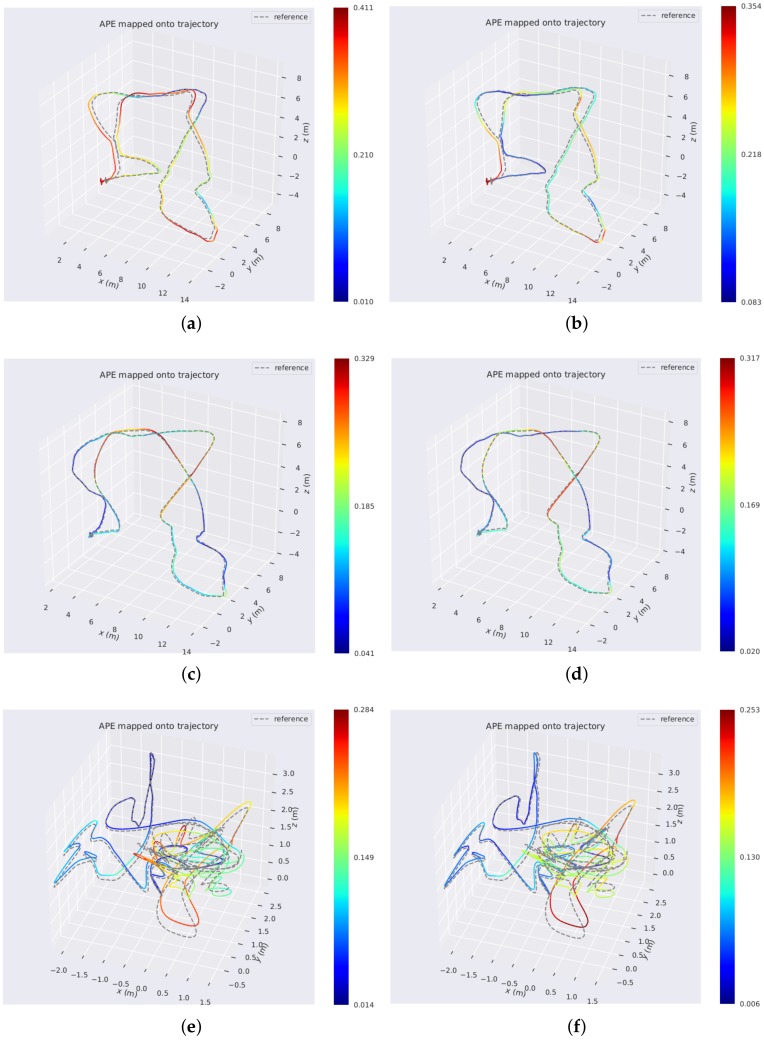
Comparison of the proposed method versus VINS-Mono. The three colorful trajectories of the left column are run with VINS-Mono on the (**a**) MH_05_difficult; (**c**) MH_04_difficult; and (**e**) V1_03_difficult sequences. The right three trajectories are the results of the proposed PL-VIO on the (**b**) MH_05_difficult; (**d**) MH_04_difficult; and (**f**) V1_03_difficult sequences. Colors encode the corresponding absolute pose errors.

**Figure 8:**
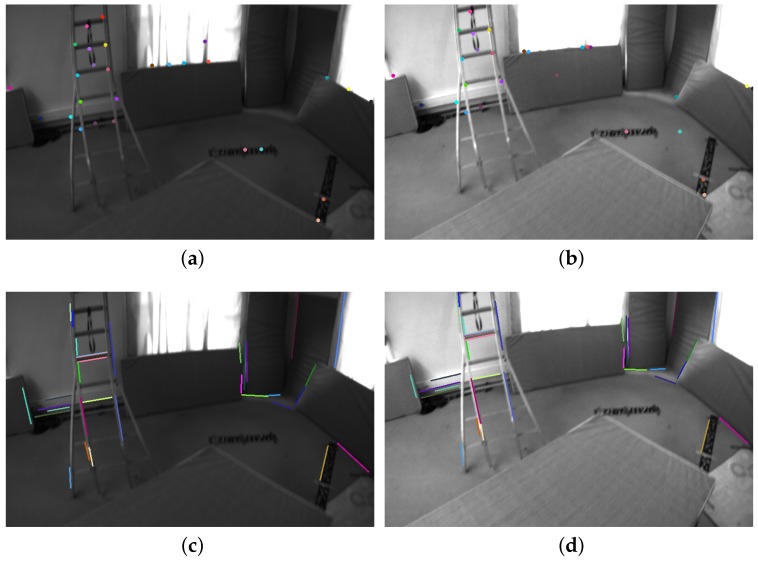
Tracked point/line features with rapid rotation in V1_03_difficult. Lighting conditions and view directions changed in successive frames. Matched point features are marked with the same color in frame (**a**,**b**). Matched line segment features are marked with the same color in frame (**c**,**d**).

Table 1 shows that our PL-VIO which jointly optimizes point and lines provided the best performance on eight sequences for the rotation, except for V1_01_easy. Our method also performed the best on six sequences for the translation, except for V1_01_easy, V1_02_medium, and V1_03_difficult. However, the difference with respect to the best results was only at the submillimeter level. The results in Table 1 show that integrating line features into VIO could improve the accuracy of motion estimation. To demonstrate the results intuitively, several heat map of trajectories estimated by PL-VIO and VINS-Mono are shown in Figure 7. The redder the color is, the larger the translation error is. Comparing the three trajectories, we came to the conclusion that PL-VIO with line features gave smaller errors than VINS-Mono when the camera was moved with rapid rotation. Furthermore, we found that these sequences with rapid rotation caused large changes in the viewing direction, and the lighting conditions are especially challenging for tracking point features [25,26,28]. As shown in Figure 8, 27 point pairs (including 10 outliers) were matched successfully, while 33 lines were matched successfully and all the matches are correct.

Besides, the computation times of different methods are listed in Table 2. The computation efficiency of filter-based ROVIO was the highest, while its accuracy was the lowest. The proposed PL-VIO system was the most time-consuming method, but its computation time was mainly restricted by the line detection and matching step. In Section 5.3, the computation times of different modules in PL-VIO are independently estimated in the V1_03_difficult sequence, and it was found that the computation efficiency of the PL-VIO system directly depended on the line detection and matching.

### 5.2. PennCOSYVIO Dataset

**Figure 9:**
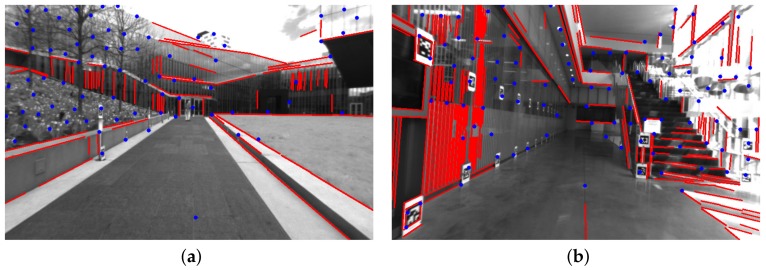
Images from PennCOSYVIO dataset. Red lines are detected lines. (**a**) Outside the glass building. (**b**) Inside the glass building.

The PennCOSYVIO dataset is a recent VIO benchmark that collects the synchronized data of a large glass building with hand-held equipment from outdoors to indoors (see Figure 9) [45]. Challenging factors include illumination changes, rapid rotations, and repetitive structures. All the intrinsic and extrinsic parameters as well as the ground truth trajectory are provided. We use data collected by the VI-sensor, which was also used in the EuRoc MAV datasets.

We compared the proposed PL-VIO with the VINS-Mono without loop closure. To evaluate fairly, the same parameters were used for PL-VIO and VINS-Mono. The same metric and evaluation tools used in Section 5.1 were employed here to evaluate the trajectories. Table 3 lists the results.

Furthermore, the evaluation tool (https://daniilidis-group.github.io/penncosyvio/) is also provided in the PennCOSYVIO dataset, and adopts two metrics, the APE and relative pose error (RPE). For RPE, it expresses the errors in percentages by dividing the value with the path length [45]. The creators of PennCOSYVIO cautiously selected the evaluation parameters, so their tool is suited for evaluating VIO approaches in this dataset. Therefore, we adopted this evaluation tool in our experiments, and the results are listed in Table 4.

From Table 3 and Table 4, it can be seen that the PL-VIO obtained the best performance for the rotation part. The APE of translation evaluated by PennCOSYVIO tool provided more details. Compared to VINS-Mono, PL-VIO gave smaller errors in the *y*-axis and *z*-axis, and a smaller error summation of the three axes. VINS-Mono obtained better performance only in the *x*-axis.

### 5.3. Computing Time

Finally, we evaluated the average execution time of our PL-VIO running at the V1_02_medium sequence because this image sequence was collected from a typical indoor scene. Table 5 shows the execution time of each block. We can see that line detection and matching, which runs at 11 Hz in the front end, is the bottleneck in terms of efficiency. State-of-the-art line detection and matching methods, such as the combination of LSD and LBD, are not satisfactory for VIO/SLAM systems. Note that our method is independent of line feature detection and matching, so improving their efficiency is beyond the scope of this paper. Marginalization in the back end is another time-consuming part. We observe that the inefficiency of marginalization is caused by the fill-in when marginalizing out features, which makes the Hessian matrix become a less sparse matrix. This problem can be potentially solved by discarding some features when performing marginalization to maintain a sparse Hessian matrix [18].

## 6. Conclusions

This paper presents the novel tightly-coupled monocular vision-inertial odometry algorithm PL-VIO, which optimizes the system states in a sliding window with both point and line features. The proposed PL-VIO system has two main modules: the front end and the back end. The front-end module is used to propagate IMU body state, and detect and match point/line features. The back-end module is used to estimate and optimize the body states. In the back-end module, a line landmark is considered as an infinite 3D spatial line and its orthonormal representation is employed to parameterize it compactly during optimization. Furthermore, all the Jacobian matrices of error terms are given in detail for solving the sliding window optimization efficiently. We also provide the evaluation results of the proposed PL-VIO as compared to three state-of-the-art monocular VIO methods including ROVIO [17], OKVIS [18], and VINS-Mono [32] on both the EuRoc dataset and PennCOSYVIO dataset. According to the analysis and results, two further conclusions are as follows:

1. The reconstructed 3D map with line features can provide geometrical information with respect to the environment, and thus semantic information could be extracted from the map. This is useful for robot navigation.
2. Line features can improve the system accuracy both for translation and rotation, especially in illumination-changing scenes. However, the line detection and matching are time-consuming and become the bottlenecks in the efficiency of the system.

In the future, we plan to improve our system by introducing the structural constraints between 3D spatial lines, such as parallel or coplanar lines in Manhattan-world scenes [48]. Geometric constraints among these lines have the potential to further improve localization precision and reduce rotation accumulation errors.

## Figures and Tables

**Table 1:** The root mean square error (RMSE) results on the several EuRoc MAV dataset. The translation (m) and rotation (rad) error are listed as follows. The numbers in bold represent the estimated trajectory is more close to the benchmark trajectory.

Seq.	ROVIO		OKVIS-Mono		VINS-Mono		PL-VIO	
	Trans.	Rot.	Trans.	Rot.	Trans.	Rot.	Trans.	Rot.
MH_02_easy	0.59075	2.21181	0.36059	0.06095	0.25663	0.04802	**0.23274**	**0.04204**
MH_03_medium	0.40709	1.92561	0.21534	0.02622	0.11239	0.02027	**0.11224**	**0.02016**
MH_04_difficult	0.88363	2.30330	0.23984	0.01943	0.15366	0.02173	**0.13942**	**0.01915**
MH_05_difficult	1.17578	2.27213	0.39644	0.01987	0.30351	0.01038	**0.25687**	**0.00892**
V1_01_easy	0.18153	2.03399	0.08583	**0.09665**	**0.05843**	0.09995	0.05916	0.09869
V1_02_medium	0.19563	1.93652	0.12207	0.04073	**0.06970**	0.03022	0.07656	**0.02871**
V1_03_difficult	0.17091	2.02069	0.19613	0.06591	0.14531	0.08021	**0.13016**	**0.04382**
V2_02_medium	0.60686	1.84458	0.18253	0.04773	0.10218	0.04558	**0.09450**	**0.04177**
V2_03_difficult	**0.18912**	1.92380	0.30513	0.07527	0.26446	0.06162	0.26085	**0.06098**

**Table 2:** Average running times of different methods on the EuRoc dataset (unit: millisecond).

Seq.	ROVIO	OKVIS-Mono	VINS-Mono	PL-VIO
MH_02_easy	15	31	63	127
MH_03_medium	15	30	62	112
MH_04_difficult	15	24	54	108
MH_05_difficult	15	27	58	102
V1_01_easy	14	26	45	93
V1_02_medium	15	23	37	86
V1_03_difficult	15	20	29	82
V2_02_medium	15	22	33	86
V2_03_difficult	15	21	27	85

**Table 3:** The RMSE of the absolute pose error (APE) for different algorithms. The numbers in bold represent the estimated trajectory is more close to the benchmark trajectory.

Algorithm	Translation Error (m)	Rotation Error (rad)
VINS-Mono	1.14690	0.04156
PL-VIO	**1.05975**	**0.03742**

**Table 4:** The results evaluated by PennCOSYVIO evaluation tool for different algorithms. The rotation errors for the APE and relative pose error (RPE) are expressed in degrees. The translation error is expressed in the *x*-axis, *y*-axis, and *z*-axis. The APE is expressed in meters, while the RPE is expressed in percentages (%). The numbers in bold represent the estimated trajectory is more close to the benchmark trajectory.

Algorithm	APE				RPE			
	*x*	*y*	*z*	Rot.	*x*	*y*	*z*	Rot.
VINS-Mono	**0.423**	0.173	0.861	2.3477	2.807	1.844	4.663	1.9337
PL-VIO	0.524	**0.070**	**0.769**	**2.0782**	**2.375**	1.844	**4.361**	**1.7350**

**Table 5:** Mean execution time of PL-VIO run with the V1_02_medium sequence.

Module	Operation	Times (ms)	Rate (Hz)	Thread ID
front end	point feature detection and matching	4	25	1
	line feature detection and matching	86	11	2
	IMU forward propagation	1	100	3
back end	nonlinear optimization	28	15	4
	marginalization	35	15	4
	feature triangulation and culling	2	15	4

## Appendix A

The IMU error state propagtion equation can be defined as [49]:

(A1) $$\left[\begin{array}{c} \delta \alpha_{b_{k+1}b_{k+1}^{\prime }}\\ \delta \theta_{b_{k+1}b_{k+1}^{\prime }}\\ \delta \beta_{b_{k+1}b_{k+1}^{\prime }}\\ \delta b_{a}^{b_{k+1}}\\ \delta b_{g}^{b_{k+1}} \end{array}\right]=F_{k}\left[\begin{array}{c} \delta \alpha_{b_{k}b_{k}^{\prime }}\\ \delta \theta_{b_{k}b_{k}^{\prime }}\\ \delta \beta_{b_{k}b_{k}^{\prime }}\\ \delta b_{a}^{b_{k}}\\ \delta b_{g}^{b_{k}} \end{array}\right]+G_{k}\left[\begin{array}{c} n_{a}^{b_{k}}\\ n_{g}^{b_{k}}\\ n_{a}^{b_{k+1}}\\ n_{g}^{b_{k+1}}\\ n_{b_{a}}\\ n_{b_{g}} \end{array}\right]$$

With

(A2) $$F_{k}=\left[\begin{array}{ccccc} I & f_{12} & I\delta t & -\frac{1}{4}\left(q_{b_{i}b_{k}}+q_{b_{i}b_{k+1}}\right)\delta t^{2} & f_{15}\\ 0 & I-{\left[\omega \right]}_{\times } & 0 & 0 & -I\delta t\\ 0 & f_{32} & I & -\frac{1}{2}\left(q_{b_{i}b_{k}}+q_{b_{i}b_{k+1}}\right)\delta t & f_{35}\\ 0 & 0 & 0 & I & 0\\ 0 & 0 & 0 & 0 & I \end{array}\right]$$

(A3) $$G_{k}=\left[\begin{array}{cccccc} \frac{1}{4}q_{b_{i}b_{k}}\delta t^{2} & g_{12} & \frac{1}{4}q_{b_{i}b_{k+1}}\delta t^{2} & g_{14} & 0 & 0\\ 0 & \frac{1}{2}\delta t & 0 & \frac{1}{2}\delta t & 0 & 0\\ \frac{1}{2}q_{b_{i}b_{k}}\delta t & g_{32} & \frac{1}{2}q_{b_{i}b_{k+1}}\delta t & g_{34} & 0 & 0\\ 0 & 0 & 0 & 0 & \delta t & 0\\ 0 & 0 & 0 & 0 & 0 & \delta t \end{array}\right]$$

(A4) $$\begin{array}{cc} f_{12} & =\frac{\partial \alpha_{b_{i}b_{k+1}}}{\partial \delta \theta_{b_{k}b_{k}^{\prime }}}=-\frac{1}{4}\left(R_{b_{i}b_{k}}{\left[a^{b_{k}}-b_{a}^{b_{k}}\right]}_{\times }\delta t^{2}+R_{b_{i}b_{k+1}}{\left[\left(a^{b_{k}}-b_{a}^{b_{k}}\right)\right]}_{\times }\left(I-{\left[\omega \right]}_{\times }\delta t\right)\delta t^{2}\right)\\ f_{32} & =\frac{\partial \beta_{b_{i}b_{k+1}}}{\partial \delta \theta_{b_{k}b_{k}^{\prime }}}=-\frac{1}{2}\left(R_{b_{i}b_{k}}{\left[a^{b_{k}}-b_{a}^{b_{k}}\right]}_{\times }\delta t+R_{b_{i}b_{k+1}}{\left[\left(a^{b_{k}}-b_{a}^{b_{k}}\right)\right]}_{\times }\left(I-{\left[\omega \right]}_{\times }\delta t\right)\delta t\right)\\ f_{15} & =\frac{\partial \alpha_{b_{i}b_{k+1}}}{\partial \delta b_{g}^{b_{k}}}=-\frac{1}{4}\left(R_{b_{i}b_{k+1}}{\left[\left(a^{b_{k}}-b_{a}^{b_{k}}\right)\right]}_{\times }\delta t^{2}\right)\left(-\delta t\right)\\ f_{35} & =\frac{\partial \beta_{b_{i}b_{k+1}}}{\partial \delta b_{g}^{b_{k}}}=-\frac{1}{2}\left(R_{b_{i}b_{k+1}}{\left[\left(a^{b_{k}}-b_{a}^{b_{k}}\right)\right]}_{\times }\delta t\right)\left(-\delta t\right)\\ g_{12} & =\frac{\partial \alpha_{b_{i}b_{k+1}}}{\partial n_{g}^{b_{k}}}=g_{14}=\frac{\partial \alpha_{b_{i}b_{k+1}}}{\partial n_{g}^{b_{k+1}}}=-\frac{1}{4}\left(R_{b_{i}b_{k+1}}{\left[\left(a^{b_{k}}-b_{a}^{b_{k}}\right)\right]}_{\times }\delta t^{2}\right)\left(\frac{1}{2}\delta t\right)\\ g_{32} & =\frac{\partial \beta_{b_{i}b_{k+1}}}{\partial n_{g}^{b_{k}}}=g_{34}=\frac{\partial \beta_{b_{i}b_{k+1}}}{\partial n_{g}^{b_{k+1}}}=-\frac{1}{2}\left(R_{b_{i}b_{k+1}}{\left[\left(a^{b_{k}}-b_{a}^{b_{k}}\right)\right]}_{\times }\delta t^{2}\right)\left(\frac{1}{2}\delta t\right) \end{array}$$

We can define the error state vector with $\eta_{k+1}={\left[\delta \alpha_{b_{k+1}b_{k+1}^{\prime }},\delta \theta_{b_{k+1}b_{k+1}^{\prime }},\delta \beta_{b_{k+1}b_{k+1}^{\prime }},\delta b_{a}^{b_{k+1}},\delta b_{g}^{b_{k+1}}\right]}^{⊤}$. The noise vector is $n_{k}={\left[n_{a}^{b_{k}},n_{g}^{b_{k}},n_{a}^{b_{k+1}},n_{g}^{b_{k+1}},n_{b_{a}},n_{b_{g}},\right]}^{⊤}$. Equation (A1) can be written in compact matrix form as:

(A5) $$\eta_{k+1}=F_{k}\eta_{k}+G_{k}n_{k}$$

The pre-integrated measurements covariance can be computed iteratively based on the linear model (A5) [14]:

(A6) $$\Sigma_{b_{i}b_{k+1}}=F_{k}\Sigma_{b_{i}b_{k}}F_{k}^{⊤}+G_{k}\Sigma_{n}G_{k}^{⊤}$$

where $\Sigma_{n}$ is the covariance of the raw IMU measurements, and the initial covariance is $\Sigma_{b_{i}b_{i}}=0_{15\times 15}$. Also we can compute the Jacobian matrix of pre-integrated measurements $z_{b_{i}b_{j}}$ with respect to error state $\eta_{i}$ iteratively with [49]:

(A7) $$J_{ik+1}=F_{k}J_{ik}$$

With $J_{ii}=I$. These Jacobian matrices given in Equation (10) can be extracted from $J_{ij}$.

## Appendix B

The Jacobian matrix of the line re-projection error respect to the orthonormal representation is:

(A8) $$\begin{array}{cc} \frac{\partial r_{l}}{\partial l} & =\left[\begin{array}{ccc} \frac{\partial r_{1}}{\partial l_{1}} & \frac{\partial r_{1}}{\partial l_{2}} & \frac{\partial r_{1}}{\partial l_{3}}\\ \frac{\partial r_{2}}{\partial l_{1}} & \frac{\partial r_{2}}{\partial l_{2}} & \frac{\partial r_{2}}{\partial l_{3}} \end{array}\right]\\ & ={\left[\begin{array}{ccc} \frac{-l_{1}s_{l}^{⊤}l}{{\left(l_{1}^{2}+l_{2}^{2}\right)}^{\left(\frac{3}{2}\right)}}+\frac{u_{s}}{{\left(l_{1}^{2}+l_{2}^{2}\right)}^{\left(\frac{1}{2}\right)}} & \frac{-l_{2}s_{l}^{⊤}l}{{\left(l_{1}^{2}+l_{2}^{2}\right)}^{\left(\frac{3}{2}\right)}}+\frac{v_{s}}{{\left(l_{1}^{2}+l_{2}^{2}\right)}^{\left(\frac{1}{2}\right)}} & \frac{1}{{\left(l_{1}^{2}+l_{2}^{2}\right)}^{\left(\frac{1}{2}\right)}}\\ \frac{-l_{1}e_{l}^{⊤}l}{{\left(l_{1}^{2}+l_{2}^{2}\right)}^{\left(\frac{3}{2}\right)}}+\frac{u_{e}}{{\left(l_{1}^{2}+l_{2}^{2}\right)}^{\left(\frac{1}{2}\right)}} & \frac{-l_{2}e_{l}^{⊤}l}{{\left(l_{1}^{2}+l_{2}^{2}\right)}^{\left(\frac{3}{2}\right)}}+\frac{v_{e}}{{\left(l_{1}^{2}+l_{2}^{2}\right)}^{\left(\frac{1}{2}\right)}} & \frac{1}{{\left(l_{1}^{2}+l_{2}^{2}\right)}^{\left(\frac{1}{2}\right)}} \end{array}\right]}_{2\times 3} \end{array}$$

(A9) $$\begin{array}{cc} \frac{\partial L_{w}}{\partial \delta O} & ={\left[\begin{array}{cccc} \frac{\partial L_{w}}{\partial u_{1}} & \frac{\partial L_{w}}{\partial u_{2}} & \frac{\partial L_{w}}{\partial w_{1}} & \frac{\partial L_{w}}{\partial w_{2}} \end{array}\right]}_{6\times 8}{\left[\begin{array}{cc} \frac{\partial u_{1}}{\partial \delta \psi } & \frac{\partial u_{1}}{\partial \delta \phi }\\ \frac{\partial u_{2}}{\partial \delta \psi } & \frac{\partial u_{2}}{\partial \delta \phi }\\ \frac{\partial w_{1}}{\partial \delta \psi } & \frac{\partial w_{1}}{\partial \delta \phi }\\ \frac{\partial w_{2}}{\partial \delta \psi } & \frac{\partial w_{2}}{\partial \delta \phi } \end{array}\right]}_{8\times 4}\\ & ={\left[\begin{array}{cccc} w_{1}I_{3\times 3} & 0_{3\times 3} & u_{1} & 0_{3\times 1}\\ 0_{3\times 3} & w_{2}I_{3\times 3} & 0_{3\times 1} & u_{2} \end{array}\right]}_{6\times 8}{\left[\begin{array}{cccc} 0 & -u_{3} & u_{2} & 0\\ u_{3} & 0 & -u_{1} & 0\\ 0 & 0 & 0 & -w_{2}\\ 0 & 0 & 0 & w_{1} \end{array}\right]}_{8\times 4}\\ & ={\left[\begin{array}{cccc} 0 & -w_{1}u_{3} & w_{1}u_{2} & -w_{2}u_{1}\\ w_{2}u_{3} & 0 & -w_{2}u_{1} & w_{1}u_{2} \end{array}\right]}_{6\times 4} \end{array}$$

To compute the line re-projection error, a spatial line in world frame *w* is transformed to the body frame *b* firstly, and then transformed to the camera frame *c* with the extrinsic parameters $T_{bc}$.

(A10) $$\begin{array}{cc} L_{c} & =T_{bc}^{-1}T_{wb}^{-1}L_{w}\\ & =T_{bc}^{-1}{\left[\begin{array}{c} R_{wb}^{⊤}\left(n^{w}+{\left[d^{w}\right]}_{\times }p_{wb}\right)\\ R_{wb}^{⊤}d^{w} \end{array}\right]}_{6\times 1} \end{array}$$

The Jacobian matrix of the line re-projection error with respect to rotation of the $i^{th}$ IMU body state is:

(A11) $$\begin{array}{cc} \frac{\partial L_{c}}{\partial \delta \theta_{bb^{\prime }}} & =T_{bc}^{-1}\left[\begin{array}{c} \frac{\partial \left(I-{\left[\delta \theta_{bb^{\prime }}\right]}_{\times }\right)R_{wb}^{⊤}\left(n^{w}+{\left[d^{w}\right]}_{\times }p_{wb}\right)}{\partial \delta \theta_{bb^{\prime }}}\\ \frac{\partial \left(I-{\left[\delta \theta_{bb^{\prime }}\right]}_{\times }\right)R_{wb}^{⊤}d^{w}}{\partial \delta \theta_{bb^{\prime }}} \end{array}\right]\\ & =T_{bc}^{-1}{\left[\begin{array}{c} {\left[R_{wb}^{⊤}\left(n^{w}+{\left[d^{w}\right]}_{\times }p_{wb}\right)\right]}_{\times }\\ R_{wb}^{⊤}d^{w}{]}_{\times } \end{array}\right]}_{6\times 3} \end{array}$$

The Jacobian matrix of the line re-projection error with respect to position of the *i*th IMU body state is as follows:

(A12) $$\begin{array}{cc} \frac{\partial L_{c}}{\partial \delta p_{bb^{\prime }}} & =T_{bc}^{-1}\left[\begin{array}{c} \frac{\partial R_{wb}^{⊤}\left(n^{w}+{\left[d^{w}\right]}_{\times }\left(p_{wb}+\delta p_{bb^{\prime }}\right)\right)}{\partial \delta p_{bb^{\prime }}}\\ \frac{\partial R_{wb}^{⊤}d^{w}}{\partial \delta p_{bb^{\prime }}} \end{array}\right]\\ & =T_{bc}^{-1}{\left[\begin{array}{c} R_{wb}^{⊤}{\left[d^{w}\right]}_{\times }\\ 0 \end{array}\right]}_{6\times 3} \end{array}$$
